# Calendar

**DOI:** 10.1155/S0962935193000651

**Published:** 1993


Mediators of Inflammation 2, 447–452 (1993)

### Isolation and function of a human endothelial cell C1q receptor

M. R. Daha[CA] L. Dunn, R. van den Berg, Y. Muizert-de Lange, A. Gerritsen and L. A. van Es

Department of Nephrology, University Hospital Leiden, P.O. Box 9600, building 1, C3-P, 2300 RC Leiden, The Netherlands

[CA] Corresponding Author

**IT has been shown previously that cultured human venous and arterial endothelial cells (EC) bind C1q in a time- and dose-dependent manner. Cultured human endothelial cells express an average number of 5.2 × $10^5$ binding sites/cell. In the present study the putative receptor for C1q (C1qR) was isolated from the membranes of 1–5 × $10^9$ human umbilical cord EC by affinity chromatography on C1q–Sepharose. During isolation, C1qR was detected by its capacity to inhibit the lysis of EAC1q in C1q-deficient serum. The eluate from C1q–Sepharose was concentrated, dialysed and subjected to QAE-A50 chromatography and subsequently to gel filtration on HPLC–TSK 3000. C1qR filtered at an apparent molecular weight of 60 kDa. Purified C1qR exhibited an apparent molecular weight of 55–62 kDa in the unreduced state and a molecular weight of 64–68 kDa in reduced form. Two IgM monoclonal antibodies (mAb) D3 and D5 were raised following immunization of mice with purified receptor preparations. Both monoclonal antibodies increased the binding of $^{125}$I-C1q to endothelial cells but F(ab')$_2$ anti-C1qR mAb inhibited the binding of $^{125}$I-C1q to EC in a dose-dependent manner. The D3 mAb recognized a band of 54–60 kDa in Western blots of membranes of human EC and polymorphonuclear leukocytes. Previously, the authors showed that C1q induces the binding of IgM-containing immune complexes to EC. Therefore, it was hypothesized that during a primary immune response generation of IgM-IC may occur, resulting in binding and activation of C1, dissociation of activated C1 by C1 inhibitor and subsequent interaction of IgM-IC bearing C1q with EC–C1qR.**

**Key words:** Complement, C1q, C1q receptor, Endothelial cells, Immune complexes, Monoclonal antibodies

### Introduction

The endothelial cell layer represents a barrier between the circulation and the vessel wall, and may play an important role in processes which mediate inflammation. The role of the vascular endothelium in the deposition of immune complexes (IC) *in vivo* is not clear. Most IC are able to trigger receptor mediated activation of various cells such as monocytes and granulocytes. This may occur via Fc receptors alone or via Fc receptors in synergy with complement receptors. Receptors for the Fcγ portion of IgG have been described on various cells,[1] and more recently the authors have shown that rat liver endothelial cells express functional receptors for the Fcγ region of IgG *in situ*.[2] Furthermore, receptors for the Fcγ region of IgG and C3b were found on *Herpes simplex* virus (HSV)-infected umbilical cord venous endothelial cells.[3] Later studies have established that these receptors are encoded by the viral genome.[4,5] The first indication that another complement receptor was present on endothelial cells, namely a receptor for C1q, was obtained by Linder[6] and further established by other.[7] The authors have described previously that human umbilical cord venous and arterial EC express substantial numbers of C1qR.[8] More insight into the binding of C1q to a variety of somatic and cultured cells and further identification of a putative C1qR has been obtained more recently.[9–12] In the present study C1qR was purified from the membranes of cultured umbilical cord EC and it appears to be closely related to C1q receptors purified from peripheral blood leukocytes[13–15] and platelets.[16]

### Materials and Methods

*Endothelial cell cultures:* Human umbilical cord vein endothelial cells (HUVEC) were isolated and cultured as described.[8,17] In short, umbilical cord veins were flushed with phosphate buffered saline and incubated with a collagenase solution (1 mg/ml,

© 1993 Rapid Communications of Oxford Ltd

Sigma, St. Louis, MO) for 20 min at 37°C. Cells were cultured on 1% gelatin treated plastic culture flasks (T75, Greiner) in M199 with Earls salts (Seromed, Biochrom KG, Berlin, Germany) supplemented with 20% heat inactivated normal human serum and endothelial cell growth factor[18] isolated from bovine hypothalamus. Cells were subcultured after trypsinization by distributing them into three new culture flasks. For the isolation of C1qR generally 1–5 × $10^9$ HUVEC were used from passage 4–7. In most cases endothelial cells obtained from three to four umbilical cords were pooled. HUVEC were routinely examined by indirect immunofluorescence employing a rabbit polyclonal anti-human WF antibody (gift of Professor R. Bertina, Leiden).

Isolation of C1qR was also performed from the membranes of the endothelial hybridoma cell line EAhy.926. This line was obtained by fusing human umbilical vein endothelial cells with the human cell line A549 (human lung carcinoma) and was maintained in culture as described previously.[19]

*Solubilization of HUVEC and EAhy.926:* Confluent layers of cells were rinsed with sterile PBS, the cells detached subsequently by incubation for 30 min at 0°C in PBS containing 10 mM EDTA, and washed three times by centrifugation at 150 × *g* and resuspension in ice-cold water containing 5 mM EDTA. Usually, 1–5 × $10^9$ cells were resuspended in 5 ml water, and frozen at −80°C. Thereafter the cells were frozen and thawed a total of five times. The resultant mixture was centrifuged for 10 min at 15 000 × *g* and the pellet containing mainly cell membranes washed three times with ice-cold PBS containing 5 mM EDTA. The washed cell membrane pellet was finally resuspended in 2 ml lysis buffer composed of 5 mM sodium phosphate, 5 mM EDTA, 150 mM NaCl, 10 mM EACA and 0.5 mM PMSF, pH 7.5 and containing 1% nonidet P40. After incubation for 60 min at 37°C with vigorous shaking, the mixture was centrifuged for 20 min at 30 000 × *g* and the supernatant dialysed against lysis buffer containing 0.1% NP40.

*Purification of C1qR:* Endothelial cell membrane lysates were loaded on a column of 4 ml Sepharose-C1q equilibrated in PBS containing 0.5 mM PMSF, 5 mM EDTA and 0.1% NP40, pH 7.5. C1q was isolated from pooled human serum as described previously[20] and 3 mg of C1q was coupled to 1 ml of packed Sepharose. After vigorous washing bound C1qR was liberated from the column using 1 M NaCl. Protein content in the fractions was measured by the Lowry method and conductivity was assessed at 4°C. C1qR was assayed in the fractions using a haemolytic assay. The fractions containing C1qR activity were pooled, dialysed against 5 mM PMSF and subjected to ion exchange chromatography on a 1.5 × 10 cm QAE–A50 Sephadex column equilibrated in dialysis buffer. Bound activity was stripped from the column with dialysis buffer containing 0.65 M NaCl. C1qR activity was pooled, freeze dried, resuspended in 250 μl PBS containing 5 mM EDTA and 0.5 mM PMSF and after filtration on millipore 0.2 μ subjected to gel filtration on TSK 3000–HPLC. Fractions of 0.3 ml/min were collected and assessed for C1qR activity.

*Assay for C1qR haemolytic activity:* Sheep erythrocytes (E) sensitized with optimal concentrations of rabbit IgG anti-E were prepared and incubated with a suboptimal concentration of C1q, and washed at 4°C with $GVB^{2+}$. To assay for C1qR haemolytic activity, tubes containing 1 × $10^7$ EAC1q in 100 μl $DGVB^{++}$ were incubated with dilutions of fractions from columns for 30 min at 0°C and thereafter 0.1 ml C1q deficient serum diluted 1/50 in $DGVB^{++}$ was added to each tube followed by incubation for another 60 min at 37°C. Percent haemolysis was determined following addition of 1.5 ml 0.15 M NaCl and centrifugation. Appropriate controls for reagent blank and input were included in each assay. The amount of C1q chosen to prepare EAC1q was such that EAC1q in C1q deficient serum caused approximately 60–70% lysis of the cells.

*Radioiodination procedures:* TSK 3000–HPLC-derived pools of C1qR were iodinated using 100 μCi $Na^{125}I$ (Amersham) as described previously.[21] Excess free $^{125}I$ was removed by passing the reaction mixture over a 0.5 × 5 cm Dowex 1-×8 column equilibrated in PBS containing 1% glycerol and 0.05% NP40.

Surface iodination of intact HUVEC or purified PMN[22] was performed with 1 × $10^7$ cells in 1 ml PBS at 22°C by addition of 0.5 mCi $Na^{125}I$, 60 μl lactoperoxidase (1 mg/ml), and three sequential additions (10 μl each) of $H_2O_2$ of 0.003% every 10–20 s. Thereafter the cells were washed three times by centrifugation and resuspension in PBS to remove free $^{125}I$, and finally solubilized in lysis buffer.

*SDS–polyacrylamide gel electrophoresis (SDS–PAGE):* SDS–PAGE was performed using 7.5% polyacrylamide gels.[23] Samples were mixed with an equal volume of 0.2 M Tris, 2% SDS, pH 8.0 with and without 10 mM 2β-mercaptoethanol and boiled for 5 min. Gels were stained with Coomassie brilliant blue, dried, and subjected to autoradiography using X-ray film.

Western blot analysis was performed as described previously.[24] Five and 10 μg samples of solubilized membranes were subjected to SDS–PAGE, blotted onto nitrocellulose, reacted with monoclonal antibodies or isotype controls, washed and bound antibodies reacted with biotinylated goat antimouse Ig, followed by incubation with streptavidin alkaline phosphatase (Zymed Laboratories Inc.) for 1 h, and detected with naphthol AS-MX phosphate (Sigma, St. Louis, MO) and Fast Red (Sigma) as substrate. Every incubation step was followed by a 15 min washing step in PBS–0.5% Tween 20.

*Monoclonal antibodies:* Spleen cells were obtained by standard techniques from Balb/c mice immunized with three weekly injections of 50 $\mu$g quantities of C1qR emulsified in complete Freund's adjuvant. The spleen cells were fused with non-secreting SP 20 myeloma cells and the fused cells selected in hypoxanthine–aminopterin–thymidine medium. Culture supernatants of cell lines were screened by ELISA for reactivity with purified C1qR from HUVEC. From each positive well, individual clones were prepared by adding 100 $\mu$l culture medium containing 3 cells/ml to microtitre plates. In this way two positive clones were selected (D3 and D5) for further analysis. Ascitis was prepared in Balb/c mice following injection of $1 \times 10^6$ cells per mouse. Limited proteolytic digestion of mouse IgM was carried out by incubation of 1 mg/ml solutions of D5 or control MoAb in 20 mM Tris, 150 mM NaCl, 20 mM cad2, pH 8 with 150 $\mu$g/ml diphenyl carbamyl chloride (DPCC, Sigma) treated trypsin (Sigma) for 5 h at 37°C. Mercaptoethanol was added to 10 mM and the solution incubated for 5 min at 37°C, followed by 15 $\mu$g/ml trypsin inhibitor (Sigma) to stop the reaction and further incubated for 5 min. Finally the mixture was made up to 50 mM iodoacetamide and left at room temperature for 10 min. After dialysis 7S fragments of IgM were obtained by gel filtration on Sepharose 4B. The 7S fragments were dialysed against sodium acetate buffer pH 3.8 and treated with 1% pepsin (w/w) for 16 h at 37°C and the $F(ab')_2$ fragments recovered after gel filtration on Sephadex G150.

*Binding studies:* The binding of $^{125}$I-C1q to EC was performed as described previously.[8] To determine the effect of D3 and D5 on binding of $^{125}$I-C1q to EC, monolayers of EC in 48-well culture wells were incubated with 100 ng $^{125}$I-C1q in RPMI–0.5% BSA alone or in the presence of increasing concentrations of purified $F(ab')_2$ D3 or D5 monoclonal antibodies. As a control a nonspecific $F(ab')_2$ from mouse IgM monoclonal antibody was used. After incubation for 2 h at 4°C, the wells were washed and cell bound radioactivity assessed following lysis of the cells with 100 $\mu$l 1N NaOH for 1 h.

### Results

The fractionation of detergent solubilized endothelial cell membranes from HUVEC by affinity chromatography on Sepharose-C1q is shown in Fig. 1. Most of the protein was found in the fall-through fractions, while only a small amount of protein emerged from the column between 11 and 14 mS. When the fractions from the column were tested in dilutions of 1:10 for C1qR function all the inhibitory activity was found to be associated with the protein peak in the gradient. Fractions 148–162 were pooled, dialysed and fractionated further on a QAE–A50 Sephadex column (Fig. 2). Very little detectable protein was found in the fall-through fractions and more than 80% of C1qR functional activity could be eluted from the QAE column with a step gradient of NaCl. The major peak of C1qR activity was associated with the main protein peak. To obtain some insight into the size of C1qR, fractions 52–57 were pooled, freeze dried and subjected to fractionation by HPLC on a TSK 3000 column. C1qR activity, associated with the only detectable protein peak, emerged from the column with an apparent molecular weight of 60 kDa. The fractions containing peak C1qR activity were pooled, freeze dried and part of it labelled with $^{125}$I and analysed by SDS–PAGE and autoradiography. Under non-reducing conditions only one major band was seen with an apparent molecular weight of 55–62 kDa. Under reducing conditions the molecular weight was between 64–68 kDa (Fig. 3).

FIG. 1. Affinity chromatography of detergent solubilized endothelial cell membranes on Sepharose–C1q. Protein content (O–O), conductivity (––) and C1q–R activity (●–●) in the fractions is depicted. Fractions 148–162 were pooled for further purification on QAE–A50.

FIG. 2. Anion-exchange chromatography of affinity purified C1qR on QAE–A50. Protein content (O–O), conductivity (––) and C1q–R activity (●–●) are shown. Fractions 52–57 were pooled for further analysis on TSK 3000–HPLC.

FIG. 3. SDS-PAGE of $^{125}$I-C1qR under reducing (R) and non-reducing (U) conditions.

**Table 1.** Inhibition of lysis of EAC1Q by HUVEC C1QR and by C1QR isolated from EAHY926 cells

| | Concentration of C1qR (μg/ml) | Lysis of EAC1q (Z)[a] | % Inhibition |
|---|---|---|---|
| HUVEC | | | |
| | 0 | 1.31 ± 0.11 | – |
| | 5 | 0.83 ± 0.10 | 36 |
| | 10 | 0.36 ± 0.09 | 72 |
| | 20 | 0.15 ± 0.06 | 92 |
| EAHy926 | | | |
| | 5 | 0.96 ± 0.11 | 26 |
| | 10 | 0.41 ± 0.08 | 68 |
| | 20 | 0.20 ± 0.06 | 85 |

[a] 1 × $10^7$ EAC1q in 100 μl DGVB$^{++}$ were incubated with dilutions of C1qR and C1q deficient human for 60 min at 37°C and subsequently assessed for lysis. Percent inhibition was determined relative to EAC1q lysis in C1q deficient serum in the absence of C1qR.

C1qR was also isolated from the membranes of EAhy.926 using the same procedure as described above for HUVEC–C1qR. Comparable results were obtained concerning the size and functional activity of C1qR. Purified C1qR isolated from either HUVEC or EAhy.96 were both able to inhibit lysis of EAC1q in a dose-dependent manner (Table 1). C1qR induces inhibition of lysis of EAC1q in C1q deficient serum by binding to EAC1q and presumably by preventing the interaction with C1r and C1s because EAC1q preincubated with C1qR, washed and subsequently exposed to C1q deficient serum at 37°C also exhibits inhibited lysis.

*Monoclonal antibodies:* Immunization of BALB/c mice with purified HUVEC–C1qR and fusion of spleen cells with Sp2/0 hybridoma cells yielded two monoclonal cell lines, D5 and E3, both secreting IgM. These two mAbs reacted only with C1qR and not with C1q or its fragments (Table 2). Western blot analysis revealed reactivity of D5 with one major molecule of approximately 60–64 kDa in membrane lysates of both HUVEC and PMN (Fig. 4). In some experiments an additional faint band was seen at 96–98 kDa, but this band was also seen sometimes with isotype control mAb. In addition some smaller molecular weight reaction products were seen around 45 kDa. Since the antigen used for immunization was purified by affinity chromatography over C1q–Sepharose we also tested whether D5 or E3 reacted with C1q, heat treated C1q, collagenous fragments of C1q or heads of C1q. No significant reactivity of either mAb with C1q or its fragments was found by ELISA.

*Effect of monoclonal antibodies against C1qR on C1qR mediated binding of $^{125}$I-C1q:* To determine whether the binding of $^{125}$I-C1q to HUVEC could be influenced by F(ab′)$_2$ fragments of D5, HUVEC monolayers of HUVEC were incubated with $^{125}$I-C1q in the absence or presence of increasing concentrations of mAb (Table 3). While 37.2% of $^{125}$I-C1q bound to HUVEC in medium alone, 20–200 μg F(ab′)$_2$ anti C1qR caused a dose-dependent inhibition of binding. Full inhibition of binding of $^{125}$I-C1q was not observed in any of the three experiments performed.

**Table 2.** Reactivity of D5 and E3 with C1qR or C1q and its fragments[a]

| Monoclonal antibody | Elisa wells coated with | | | |
|---|---|---|---|---|
| | C1qR | C1q | C1q heads | C1q tails |
| D5 | 2.23 ± 0.14* | 0.097 ± 0.013 | 0.099 ± 0.014 | 0.112 ± 0.014 |
| E3 | 1.78 ± 0.18 | 0.077 ± 0.009 | 0.087 ± 0.007 | 0.113 ± 0.026 |
| Isotype control | 0.098 ± 0.007 | 0.093 ± 0.012 | 0.112 ± 0.037 | 0.088 ± 0.041 |

[a] Elisa wells were coated with 1 μg/ml agent, washed and reacted with mAbs at a dilution of 1/500 in triplicate. Washed and bound antibody was detected with goat antimouse Ig-PO.
* Mean OD492 ± 2 S.D. of of three wells.

FIG. 4. Western blot analysis of monoclonal antibody against C1q–R. Five (left lanes) or 10 μg lysates of endothelial cell membranes (A) or PMN membranes (B) were separated by electrophoresis, blotted onto nitrocellulose and analysed for reactivity with D5–anti-C1qR.

### Discussion

The present study extends the previous observations[8] that human umbilical vein endothelial cells express a C1q receptor that has identity or is closely related to C1qR described on lymphocytes.[9,10,14] C1qR was isolated by affinity chromatography on C1q–Sepharose followed by further purification on QAE–A50 and TSK 3000–HPLC and detected during the isolation procedure using inhibition of lysis of EAC1q in C1q deficient serum. The first step yielded material which was reasonably pure but minor contaminants were mainly removed in the QAE–A50 step. The purified C1qR filtered with an apparent molecular weight of 60 kDa on TSK-3000. Although the molecular weights for lymphocyte C1qR have been reported to be in the range from 56–70 kDa,[25] these differences probably reflect the different percentages of acrylamide used for SDS–PAGE.

Monoclonal antibodies against the purified endothelial cell C1qR were raised. These monoclonal antibodies reacted with purified C1qR from HUVEC and from EAhy.926 cells. The purified HUVEC C1qR was also shown to react with a polyclonal antibody (kindly donated by Dr R. B. Sim, Oxford) raised against the B-cell C1qR. On the other hand it was found that while the D5 mAb reacted also with B-cells and polymorphonuclear leukocytes (PMN), the E3 mAb only reacts with EC and PMN and not with B-cells, suggesting differences in the epitopes of endothelial cell C1qR and B-cell C1qR. On the other hand $NH_2$-terminal amino acid sequence analysis of the first fourteen amino acids did not show any differences with the recently reported sequence of C1qR.[25] Further studies are needed to elucidate these differences. The D5 and E3 mAbs both recognized one major band in membrane lysates of both HUVEC and PMN. The size of C1qR from both these cell types was well within the reported range size of C1qR.[26] While the D5 mAb was able to inhibit binding of $^{125}$I-C1q to EC the E3 mAb was much weaker in this respect.

The results described in Table 1 indicate that endothelial cell C1qR is able to interact with immune-complex-bound C1q and prevent lysis of EA-C1q in C1q deficient serum, suggesting that assembly of an intact C1 on EAC1q is prevented. This mechanism may be of importance *in vivo* to regulate the degree of C1 activation in an early phase of the immune response. In addition during a primary immune response mainly IgM antibodies are generated. There are no known cellular 19S IgM receptors on human phagocytic cells, but by binding and activation of C1, and subsequent removal of activated C1r and C1s from the IgM-immune complex-bound C1q, these types of immune complexes may be trapped very rapidly on vascular endothelial cells via C1qR, which, in turn, may ingest these complexes, and prevent further systemic immune complex-mediated inflammatory responses.

**Table 3.** Inhibition of binding of $^{125}$I-C1q to endothelial cells by F(ab')$_2$ anti-C1qR

| Reagent | Percent bound | % Inhibition |
|---|---|---|
| $^{125}$I-C1q | 37.2 ± 4.8 | – |
| $^{125}$I-C1q + 20 μg anti-C1qR | 31.3 ± 4.7 | 16.9 |
| $^{125}$I-C1q + 100 μg anti-C1qR | 21.4 ± 5.1 | 32.5 |
| $^{125}$I-C1q + 200 μg anti-C1qR | 11.3 ± 3.7 | 69.3 |

10 000 cpm of $^{125}$I-C1q were used per well.

### References

1. Van de Winkel JGJ, Anderson CL. Biology of human immunoglobulin G Fc receptors. *J. Leukocyte Biol* 1991; **49:** 511.
2. Bogers WMJM, van Rooijen N, Janssen DJ, van Es LA, Daha MR. Complement enhances the elimination of soluble aggregates of IgG by rat liver endothelial cells *in vivo*. *Eur J Immunol* 1993; **23:** 433.
3. Cines DB, Lyss AP, Bina M, Corkey R, Kefalides NA, Friedman HM. Fc-and C3 receptors induced by *Herpes simplex* virus on cultured human endothelial cells. *J Clin Invest* 1982; **69:** 123.
4. Para JF, Goldstein L, Speak PG. Similarities and differences in the Fc-binding glycoprotein (gE) of *Herpes simplex* virus types 1 and 2 and tentative mapping of the viral gene for this glycoprotein. *J Virol* 1982; **41:** 137.
5. Smiley ML, Hoxil JA, Friedman H. *Herpes simplex* virus type 1 infection of endothelial, epithelial and fibroblast cells induces a receptor for C3b. *J Immunol* 1985; **134:** 2673.
6. Linder E. Binding of C1q and complement activation by vascular endothelium. *J Immunol* 1981; **126:** 648.
7. Andrews BS, Schadforth M, Cunningham P, David IV JS. Demonstration of a C1q receptor on the surface of human endothelial cells. *J Immunol* 1981; **127:** 1075.
8. Daha MR, Miltenburg AMM, Hiemstra PS, Klar-Mohammad N, van Es LA, van Hinsbergh VWM. The complement subcomponent C1q mediates binding of immune complexes and aggregates to endothelial cells *in vitro*. *Eur J Immunol* 1988; **18:** 783.
9. Tenner A. C1q interaction with cell surface receptors. *Behring Inst Mitt* 1989; **84:** 220.

10. Ghebrihiwet B. Functions associated with the C1q receptor. *Behring Inst Mitt* 1989; **84:** 204.
11. Erdei A, Reid KBM. Characterization of C1q binding material released from the membrane of Raji and U937 cells by limited proteolysis with trypsin. *Biochem J* 1988; **255:** 493.
12. Malhotra R, Thiel S, Reid KBM, Sim RB. Human leukocyte C1q receptor binds on the soluble proteins with collagen domains. *J Exp Med* 1990; **172:** 955.
13. Ghebrihiwet B. C1q receptor. *Methods Enzymol* 1987; **150:** 558.
14. Malhotra R, Sim RB. Chemical and hydrodynamic characterization of the human leukocyte receptor for complement component C1q. *Biochem J* 1989; **262:** 625.
15. Erdei A, Reid KBM. The C1q receptor. *Mol Immunol* 1988; 1067.
16. Peerschke FIB, Ghebriwet B. Platelet C1q receptor interactions with collagen- and C1q-coated surfaces. *J Immunol* 1990; **145:** 2984.
17. Jaffe EA, Nachman RL, Becker CG, Minich CR. Culture of human endothelial cells derived from umbilical veins. *J Clin Invest* 1973; **52:** 2745.
18. Maciag T, Cerun dolo J, Isley S, Kelby PR, Bordwell C. An endothelial cell growth factor from bovine hypthalamus: identification and partial characterization. *Proc Natl Acad Sci USA* 1979; **76:** 5674.
19. Edgell CS, MacDonald CC, Graham JB. Permanent cell line expressing human factor VIII-related antigen established by hybridization. *Proc Natl Acad Sci USA* 1983; **80:** 3734.
20. Daha MR, Klar N, Hoekzema R, van Es LA. Enhanced Ig production by human peripheral lymphocytes induced by aggregates. *J Immunol* 1990; **144:** 1227.
21. Thorell JL, Larson I. Lactoperoxidase coupled to polyacrylamide for radio-iodination of proteins to high specific activity. *Immunochemistry* 1974; **224:** 203.
22. Böyum A. Isolation of leukocytes from human blood. Further observations: methyl cellulose, dextran and ficoll as erythrocyte aggregating agents. *Scand J Clin Lab Invest* 1968; **21 Suppl 97:** 77.
23. Laemmli UK. Cleavage of the structural proteins during the assembly of the head of bacteriophage T4. *Nature (Lond.)* 1970; **227:** 680.
24. Van der Zee JM, Heurkens AHM, van der Voort EAM, Daha MR, Breedveld FC. Characterization of anti-endothelial antibodies in patients with rheumatoid arithritis complicated by vasculitis. *Clin Exp Rheumatol* 1991; **9:** 589.
25. Peerschke EIB, Malhotra R, Ghebrehiwet B, Reid KBM, Willis AC, Sim RB. Isolation of a human endothelial cell C1q receptor. *J Leuk Biol* 1993; **53:** 179.
26. Malhotra R, Willis AC, Jensenius JC, Jackson J, Sim RB. Structure and homology of human C1q receptor (collectin receptor). *Immunology* 1993; **78:** 341:348.

ACKNOWLEDGEMENT. The authors thank Evert Heemskerk for the isolation of C1q.

**Received 18 August 1993; accepted 7 October 1993**
